# Reinforcement Strains in Reinforced Concrete Tensile Members Recorded by Strain Gauges and FBG Sensors: Experimental and Numerical Analysis

**DOI:** 10.3390/s19010200

**Published:** 2019-01-07

**Authors:** Gintaris Kaklauskas, Aleksandr Sokolov, Regimantas Ramanauskas, Ronaldas Jakubovskis

**Affiliations:** 1 Department of Reinforced Concrete Structures and Geotechnics, Vilnius Gediminas Technical University, Sauletekio ave. 11, LT-10223 Vilnius, Lithuania; regimantas.ramanauskas@vgtu.lt (R.R.); ronaldas.jakubovskis@vgtu.lt (R.J.); 2 Research Laboratory of Innovative Building Structures, Vilnius Gediminas Technical University, Sauletekio ave. 11, LT-10223 Vilnius, Lithuania; aleksandr.sokolov@vgtu.lt

**Keywords:** Reinforced concrete, tensile elements, strain distribution, fibre Bragg grating, strain gauge, crack spacing

## Abstract

Experimental and numerical studies have been carried out on reinforced concrete (RC) short tensile specimens. Double pull-out tests employed rectangular RC elements of a length determined not to yield any additional primary cracks. Tests were carried out with tensor strain gauges installed within a specially modified reinforcement bar and, alternatively, with fibre Bragg grating based optical sensors. The aim of this paper is to analyse the different experimental setups regarding obtaining more accurate and reliable reinforcement strain distribution data. Furthermore, reinforcement strain profiles obtained numerically using the stress transfer approach and the Model Code 2010 provided bond-slip model were compared against the experimental results. Accurate knowledge of the relation between the concrete and the embedded reinforcement is necessary and lacking to this day for less scattered and reliable prediction of cracking behaviour of RC elements. The presented experimental strain values enable future research on bond interaction. In addition, few double pull-out test results are published when compared to ordinary bond tests of single pull-out tests with embedded reinforcement. The authors summarize the comparison with observations on experimental setups and discuss the findings.

## 1. Introduction

The characteristics defining reinforced concrete (RC) are more intricate than for other structural materials, as this most common building material is highly nonlinear. This nonlinearity is clearly expressed in the various complicated relations between the material, geometrical properties when it comes to predicting the structural behaviour of RC and particularly when serviceability analysis is in question. Besides the requirement for structures to carry design loads, the lifespan of the structure must be ensured through satisfaction of serviceability criteria. Among these criteria, the most imperative one for concrete is related to cracking, as it has direct influence not only on the appearance of the structure, but also impacts the design capacity as well.

The cracking phenomena is studied to this day as the approaches and equations that are available in published research [1,2,3,4] and design codes still provide very dispersed results. One common principle behind many of the approaches is the aspect of concrete and reinforcement interaction or otherwise called the bond behaviour. Classical approaches [5,6,7] either consider perfect interaction or simplified constant bond relations. Modern research has expanded on the subject over time and has proposed various equations for estimating the bond stress in the boundary layers of the reinforcement and concrete that are dependent on slippage [8,9]. When longitudinal displacements are different between the reinforcement and concrete at a specific section, the quantitative expression of this value is called slip. The bond-slip relationship is the fundamental part of the versatile approach named as the bond stress-transfer, permitting prediction of the crack spacing and width as well as the reinforcement and concrete strain distribution within an element, which in turn allows representation of the averaged deformation behaviour of RC elements. In contrast, the current design code methods of Eurocode 2 [10] and Model Code 2010 [11] employ the mean strain approach that is limited to the prediction of average deformation (displacement, deflection, etc.) behaviour of structural RC members. To progress to more accurate prediction of the cracking phenomena, further studies are needed into the bond-stress characteristics.

The most common experimental techniques to study the interaction of reinforcement and concrete are based on the measured force-displacement relationships of a reinforcing bar pulled out from the concrete (pull-out, beam, or beam end tests). In such tests, average bond stress distribution in the anchorage length as a function of the relative displacement (or slip) may be obtained. However, a number of complex and difficult to deal with side effects influence the obtained results, such as non-uniform bond stress distribution within the anchorage length, compressive stress fields arising from the supports, structural deformation of the specimen, etc. [12]. In this respect, standard pull-out tests are more suitable to study differences in the bond properties of different reinforcing bars rather than establishing precise bond-slip relationships at the serviceability limit state.

An alternative approach to study reinforcement and concrete interaction is based on the strain recordings of the reinforcement embedded into the concrete. In such tests, the bond slip behaviour of reinforcement is calculated from the obtained reinforcement strain curves. Although such a test set-up is rather complex, it reflects the phenomenon of force transfer from reinforcement to concrete and is suitable for accurate study of the bond mechanics and development of bond-slip models [13].

The accurate measurement of the reinforcement strain distribution has become possible with the development of electrical strain gauges of a short measurement base (2–10 mm). In the early attempts to measure strains along the reinforcement, the strain gauges were glued directly on the surface of bars. However, such a simplistic approach has a significant drawback due to a significant reduction of the effective bond area, thus making the results of bond stress analysis unreliable. To eliminate the latter effect as well as the influence of waterproof treatment and wiring around the rebar on bond properties, Mains [14] developed a special technique of strain measurement inside the bar. It was implemented by cutting the reinforcement in two halves, then milling the groove along the bars and gluing the gauges inside. The groove also provided space for wiring, making sure that the glued halves of the reinforcement had the appearance of a normal bar with the same bond properties. Such a technique was later used by Bresler and Bertero [15], who analysed the bond distribution in tensile RC prisms under repeated loads. Houde [16] used this technique to determine the bond stiffness of the reinforcement to concrete. Scott and Gill [13] made further improvements of the internal strain measurement technique: They experimentally determined the strain distribution in relatively long (up to 1200 mm) RC ties reinforced with 20 and 12 mm bars. The main focus by Kankam [17] was on short (200 mm length) specimens representing a fragment of the RC tensile element between two consecutive cracks. He derived bond-slip relationships from the measured steel strain profiles. Following the pioneering tests by Scott and Gill [13], Wenkenbach [18] reported the measured strain distribution in members with large diameter (up to 50 mm) bars. More recently, internally instrumented bars were used by Masukawa [19], who analysed bond deterioration due to the corrosion of steel.

Internally glued electrical strain gauges provide precise local strain values with non-affected surface bond properties, and were used in a number of the experimental programs mentioned above. However, such tests are labour-intensive and time consuming. Moreover, the number of measuring points is limited due to the space required to accommodate wiring and strain gauges. At the end of the 20th century, as a promising alternative of fibre optics sensors were applied in structural engineering [20]. Compared to electrical strain gauges, the optical fibre sensing technique is much more compact, requires less wiring, and has an option of multiple finely spaced sensors. Its efficiency of measuring the steel strain distribution in RC beams was demonstrated in the experimental study by Kenel et al. [21]. An optical fibre with a total of 286 Bragg gratings was epoxy glued in a 10 mm diameter bar, having a 1 mm wide and 1 mm deep groove milled on the surface of the bar. Such a groove, being sufficient to install and protect the optical fibre, had a rather negligible influence on the bond properties. A large number of FBG sensors spaced at 10.4 mm intervals allowed the obtaining of the smooth steel strain curve up to 0.028, a strain value which is far beyond the yielding point of reinforcement.

The current study presents both the tensor strain gauge and the optical FBG strain gauge experiments in a combined investigation of reinforcement strain distribution within the RC elements. The RC member lengths were carefully chosen to provide as much length to install sensors as possible while ensuring that the length is short enough to not induce new cracks during higher loading stages. Such a specimen length arrangement permits investigation of the interaction between concrete and the reinforcement bar at the stabilized crack stage. Experimentally obtained load-strain diagrams are presented along with numerical results based on the partial interaction approach and Model Code 2010 bond stress equation. The authors’ insights on the different experimental setups and findings from the numerical comparison are summarized in the discussion and conclusions sections.

## 2. Testing Methods

### 2.1. Specimens

The experiments were carried out on short RC prisms with an embedded reinforcement steel bar in the middle of the 150 × 150 mm section. The specimen dimensions were selected based on several key conditions. First, to increase the number of strain gauges and FBG sensors that can be installed within the investigated RC elements, their respective lengths had to be maximized. The second condition was to minimize the risk of new crack formation during the loading of the specimens, hence the RC prisms had to be short. Ideally, the RC specimen length to satisfy both conditions would be one defined by the mean crack spacing, *s_rm_*, of the chosen configuration. A new approach has previously been presented by G. Kaklauskas et al. [22] for the prediction of the mean crack spacing value depending on only a few key variables, such as the diameter of the embedded bar and the reinforcement ratio of the element. Applying the principles for the case of a Ø20 mm reinforcement bar and 150 × 150 mm concrete section, with the respective reinforcement ratio of ~1.4%, this gives an average crack spacing value of *s_rm_* ~ 250mm. Therefore, the lengths of the test specimens were taken accordingly. Under such an arrangement of the specimen length, the current study is dedicated to the investigation of the interaction between concrete and the reinforcement bar at the stabilized crack stage. The preparations of the rebars for the experiments are presented in the experimental setup sections further in the article.

### 2.2. Material Properties

The specimens were cast from ordinary Portland cement, using natural pit sand of the 0/4 mm grade and gravel grades of 4/8 and 8/11 mm. The exact ratio of cement to sand and to gravel were 1:2.6:3.05, respectively. The water to cement ratio (w/c) was 0.45. In addition, 2.48 kg/m^3^ of the additive PowerFlow 3100 (0.75%) was used. Mechanical characteristics of the specimens were obtained from standard concrete cylindrical (Ø150 × H300) sample tests. The tests found the specimen concrete strength to be equal to 45.5 MPa and 36.8 MPa for the strain gauge and FBG sensor experimental setups, respectively. It should be noted that the FBG specimen was cured by wrapping the reinforced concrete prism in wet cloths. Identical grade steel reinforcement bars were used, both bars were of S500 grade steel. Uniaxial tests were used to determine the modulus of elasticity of the Ø20 mm bars, resulting in 204 GPa. Summarized specimen specifications are provided in Table 1.

### 2.3. Strain Guage Experimental Setup

**Figure 1:**
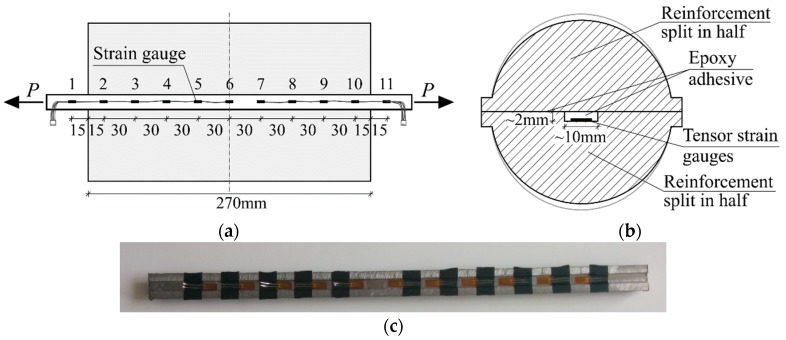
Installation and spacing of strain gauges: (**a**) Longitudinal layout; (**b**) sectional view of the reinforcement bar; and (**c**) strain gauges within one half of the reinforcement bar.

The installation of the strain gauges inside the reinforcement bar required the bars to be cut in half. A milling machine was used for this purpose to modify two distinct bars to obtain two separate halves for the new bar, with one bar also having a small longitudinal groove along the entire length to hold the mentioned strain gauges and wiring. The width and height of the groove was 10 mm and 2 mm, respectively. While the bar diameter was Ø20 mm, the authors took account of the reduction of the sectional area of the bar in comparisons of the experimental and numerical results. Two sets of gauges, one with five and the other with six gauges, were then glued to the reinforcement, with wiring carefully guided out from both ends of the bar, see Figure 1a,c. It is crucial to make sure the wiring is not in contact with the metal bar or the testing equipment afterwards as this will greatly skew or completely invalidate the gathered strain data. The 10 mm width gauges were placed with a 30 mm longitudinal spacing between their centres. Afterwards, the remaining sides of the steel bar were adhered back together with epoxy glue, as shown in Figure 1b. 

### 2.4. Fiber Bragg Grating Experimental Setup

Fibre Bragg Grating optical strain gauges are based on the principle of changes in the wavelength of the transmitted light waves as it passes through the fibre [23]. When the light wave interacts with the reflection points making up one grating, certain wavelengths will be reflected if they match the distance between these reflection points, hence the resulting wave at the other end will be transformed accordingly. These changes allow for the variation of the Bragg wavelength to be established and measure strains.

**Figure 2:**
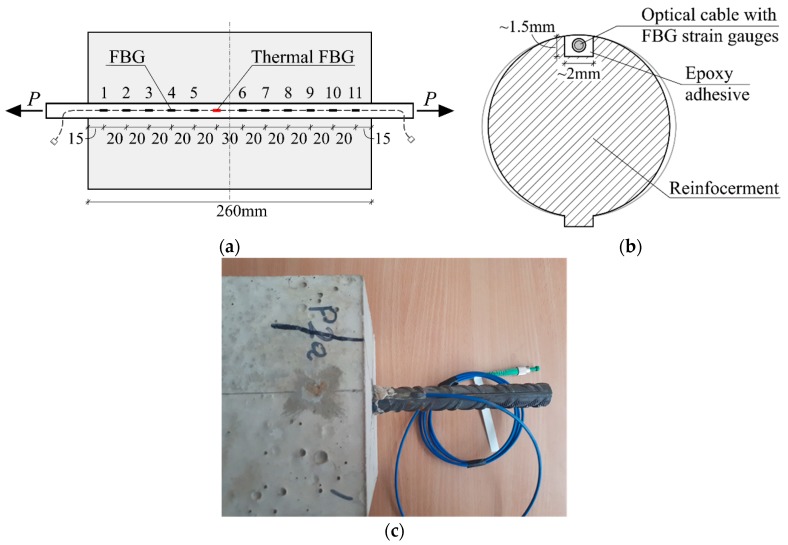
Installation and spacing of fiber Bragg grating sensors: (**a**) Longitudinal layout; (**b**) sectional view of the reinforcement bar; (**c**) enlarged view of a fragment of the test specimen.

As opposed to the tensor strain gauge specimen preparation, the reinforcement bar was not required to be divided longitudinally. However, the Ø0.9 mm optical cable was installed on the outside of the bar inside a shallow groove of around a 2 mm width and 1.5 mm depth, see Figure 2. Fixing of the optical cable to the steel bar was ensured in the same way as the tensor strain gauges, with epoxy adhesive. Optical FBG strain gauges were organized in two sets of 6 FBGs 20 mm apart from each other with the exception in the middle, where a 30 mm distance separated the two sets of sensors. One of the FBG sensors was used as a thermal calibration sensor, required to correctly evaluate readings from the optical strain gauges as the gratings are sensitive to both strain and temperature changes.

The minimal invasion into the bar’s geometry allows the attainment of the bond characteristics very closer to the real ones. In addition, more accurate load-strain data of the reinforcement bar can be recorded as a lesser reinforcement bar area is lost compared to the previous method. Furthermore, this technique allows for small diameter bars to be used as the reduced section will have less impact on the result comprehension. Whereas, the previous method might not be feasible for small bars due to a significant loss of section and a lack of the remaining sectional area to glue the bars back together. 

One specific aspect of the different location of sensors within the cross-section should be mentioned. As was said above, the strain gauges were placed close to the central axis of the reinforcement whereas the FBG sensors were located near the surface of the bar. For the reinforcement bars having a rather low modulus of elasticity (such as the FRP bars), the bond effect might result in uneven distribution of strains within the reinforcement bar. In the bars of larger diameters, external strains might exceed the ones obtained in the central part of the bar. However, for the steel bars considered in this study, the above effect should be negligible. 

### 2.5. Testing Procedure

**Figure 3:**
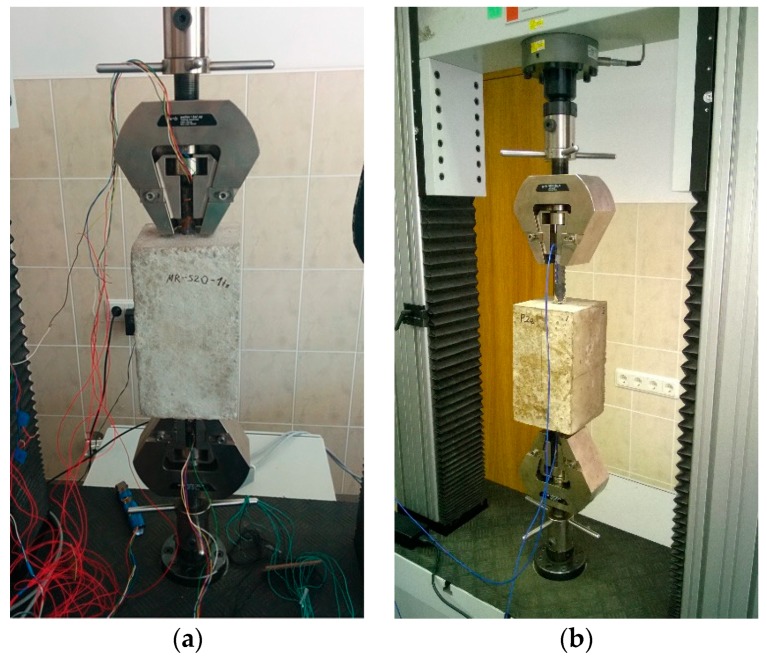
Experimental setups of: (**a**) Tensor strain gauge test and (**b**) FBG sensor test.

Both specimens were tested using the same machine and the procedure was identical. Loading increments were applied in a displacement-controlled manner, using an electromechanical testing machine, LFM100, with digital controls and DION STAT control and a data acquisition system, by pulling on the protruding reinforcement bars. The chosen loading rate was 0.5 mm/min for the tensor strain gauge specimen and 0.4 mm/min for the FBG test. Laboratory conditions were similar for both experiments, the temperature was maintained at 18.5 °C, and the relative humidity at 78%. The testing machine has an upper limit of 100 kN of applied load, therefore, the reported test results are up to 90 kN of applied load. In the case of the FBG experiment, data recording was carried out through the Fibre Sensing BraggMETER optical interrogator to interpret wavelengths that pass through the fibre and the gratings. Photos of the experimental setup are presented in Figure 3.

## 3. Results of the Experiments

**Figure 4:**
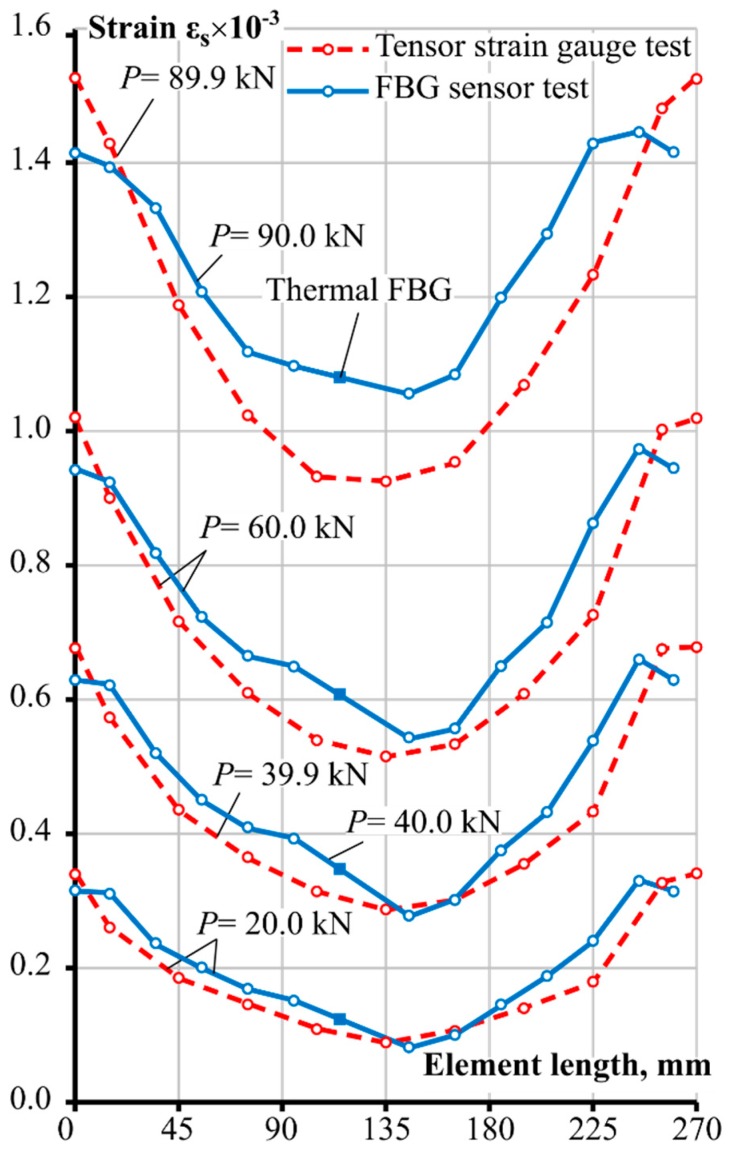
Experimental reinforcement strain distributions along the steel bars at key loadings steps.

The key results obtained from this experimental study were the reinforcement strain values at varying loading levels for each strain gauge position. The obtained data enabled graphing of the spatial changes of the strain distribution along the length of the reinforcement. Results from both experimental tests are presented in Figure 4 at four loading intervals. It can be clearly seen that at higher loading, the strain profile gradient is getting steeper. Bond characteristics have a direct influence on such an outcome, as with increased loading, i.e., the strain rate, bond stress increases. A more detailed explanation is given in the numerical analysis and discussion section of the bond interaction behaviour. Furthermore, both tests displayed that the strain values flatten out towards the end of the reinforcement bar. This could be due to some issues with the installation of the reinforcement bar between the clamps in the testing machine, however, the FBG strain gauge was fully inside the concrete part of the RC prism. G. Kaklauskas has previously proposed the zones around the normal cracks to have damaged bonds between the bar and the concrete of the RC element, and hence called these areas the debonding zones [24]. Whether this phenomenon seen in Figure 4 can be attributed to the debonding behaviour, the authors cannot be certain, as more samples would be needed to make a reliable assertion.

The thermal calibration FBG sensor was located near the middle of the element, hence strain data was not recorded at that section as defined by the long straight section of the Figure 4 chart, where a solid square marks the exact location of the thermal FBG. In addition, the authors would like to note that the end values of the bar strains of the FBG specimen were estimated based on the reinforcement elasticity modulus and the applied loading of the machine, as the optical strain gauges were 15 mm away from the edges.

## 4. Numerical Analysis and Discussion

**Figure 5:**
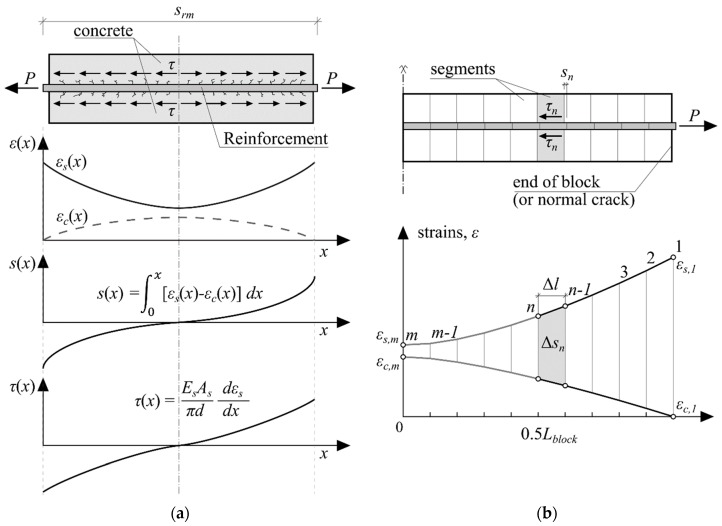
Stress transfer approach explained through (**a**) strain, slip, and bond stress variations along the reinforced concrete prism; (**b**) numerical iterative procedure as defined for half an element.

To compare the results against the Model Code 2010 prediction, numerical analysis based on the stress transfer approach was carried out. The approach enables the estimation of the strain variation within a reinforced concrete element, shown in Figure 5a, therefore, it has an inherent ability to model the cracks directly with their respective induced strain changes. The method considers the strains to be directly governed by the bond-stress between the bar and the concrete at the boundary between the two. Therefore, a significant advantage is the necessity of only the bond-slip model to carry out the analysis, as it directly relates the bond stress to the strains of the concrete and reinforcement through the slip value. In this paper, an established Model Code 2010 bond-slip model was implemented:

(1) $$\tau_{0}=\tau_{\max}{\left(s/s_{1}\right)}^{\alpha }\;\mathrm{for}\;0\leq s<s_{1}$$

where *τ*_0_ is the bond stress; *τ*_max_ is the maximum bond stress; *s* is the slip; *s*_1_ is taken as 1 mm for the case of a pull-out and good bond condition; and coefficient *α* is taken as 0.4 accordingly. For the same conditions, *τ*_max_ is equal to:

(2) $$\tau_{\max}=2.5\sqrt{f_{ck}},$$

where *f_ck_* is the characteristic concrete compressive strength. While the Model Code 2010 has extensions for cases when the slip, *s*, exceeds *s*_1_, the scenario is highly unlikely in the cases when the reinforcement stress is below the yielding stress, as was the case in these experiments as well.

To obtain the solution, a numerical iterative scheme was chosen as shown in Figure 5b. The short RC prism was divided into multiple segments, Δ*l*. Calculations were performed on every segment iteratively from the location of the normal crack, or in this case, the end of the element. The iterative procedure performed not from the middle of the RC block has the advantage of known reinforcement and concrete strain values, but lacks the knowledge of the slip value. The reinforcement strain was easily obtained from the applied load, which was known from the displacement-controlled testing on the Ø20 mm steel bar reinforced RC specimens. Concrete strains were taken as zero, since strains were yet to be transmitted from the steel bar to it at the ends of the element. Bond stress values were easily derived from the Model Code 2010 bond-slip model. However, the slip value remains unknown and was determined iteratively in this study.

The experimental testing machine was limited to applying a maximum of 100 kN of load, which led to a maximum of 30 MPa induced stress in the Ø20 mm steel bars. Consequently, no normal cracks formed in the specimens. This allows the application of the stress-transfer method without the need to consider strain redistribution that occurs the moment a normal crack opens. Matlab was used to perform the iterative calculations.

The transmitted tensile load, *P*, is equal to the sum of the axial forces present in the concrete and reinforcement at every segment:

(3) $$P=N_{s,n}+N_{c,n}=\varepsilon_{s,n}E_{s}A_{s}+\varepsilon_{c,n}E_{c}A_{c},$$

where *N_s,n_*, *ε_s,n_*, *E_s_*, and *A_s_* are the reinforcement axial force, strain, modulus of elasticity, and area, respectively; and, accordingly, the values for the concrete part are *N_c,n_*, *ε_c,n_*, *E_c_*, and *A_c_*; the underscore letter, *n*, denotes the current section under investigation.

As per Figure 5b, the strain value of the next segment was estimated as follows:

(4) $$\varepsilon_{s,n}=\varepsilon_{s,n-1}-\frac{\tau_{n}+\tau_{n-1}}{2}\cdot \frac{\pi d\cdot \Delta l}{E_{s}A_{s}},$$

where *ε_s,n_*_−1_ is the reinforcement strain value at the previous section; *τ_n_* and *τ_n_*_−1_ are the bond stress values for the current and previous sections, respectively; *d* is the diameter of the bar; and Δ*l* is the length of the section.

As bond values were unknown in the investigated section, it could be initially assumed to be equal to the previous section, *τ_n_* = *τ_n_*_−1_, and, after establishing the bond stress value, iterated again until the change in the *τ_n_* became infinitesimal. Concrete strains were then established by rearranging Equation (3) into:

(5) $$\varepsilon_{c,n}=\frac{P-\varepsilon_{s,n}E_{s}A_{s}}{E_{c}A_{c}},$$

with both strain values known at the current and previous segments, the change in slip between the two segments could now be found from:

(6) $$\Delta s_{n}=\Delta l\left(\frac{\varepsilon_{s,n}+\varepsilon_{s,n-1}}{2}-\frac{\varepsilon_{c,n}+\varepsilon_{c,n-1}}{2}\right),$$

and, thus, the slip value of the section was obtained by subtracting the change in slip from the actual slip of the previous segment, *n* − 1:

(7) $$s_{n}=s_{n-1}-\Delta s_{n},$$

convergence was achieved after iterating through all segments and reaching the middle of the RC element and the difference between the initial guessed slip value at the end of the element and the sum of all Δ*s* approached zero, within a set tolerance:

(8) $$s-\sum_{n=1}^{m}\Delta s_{n}\to 0,$$

**Figure 6:**
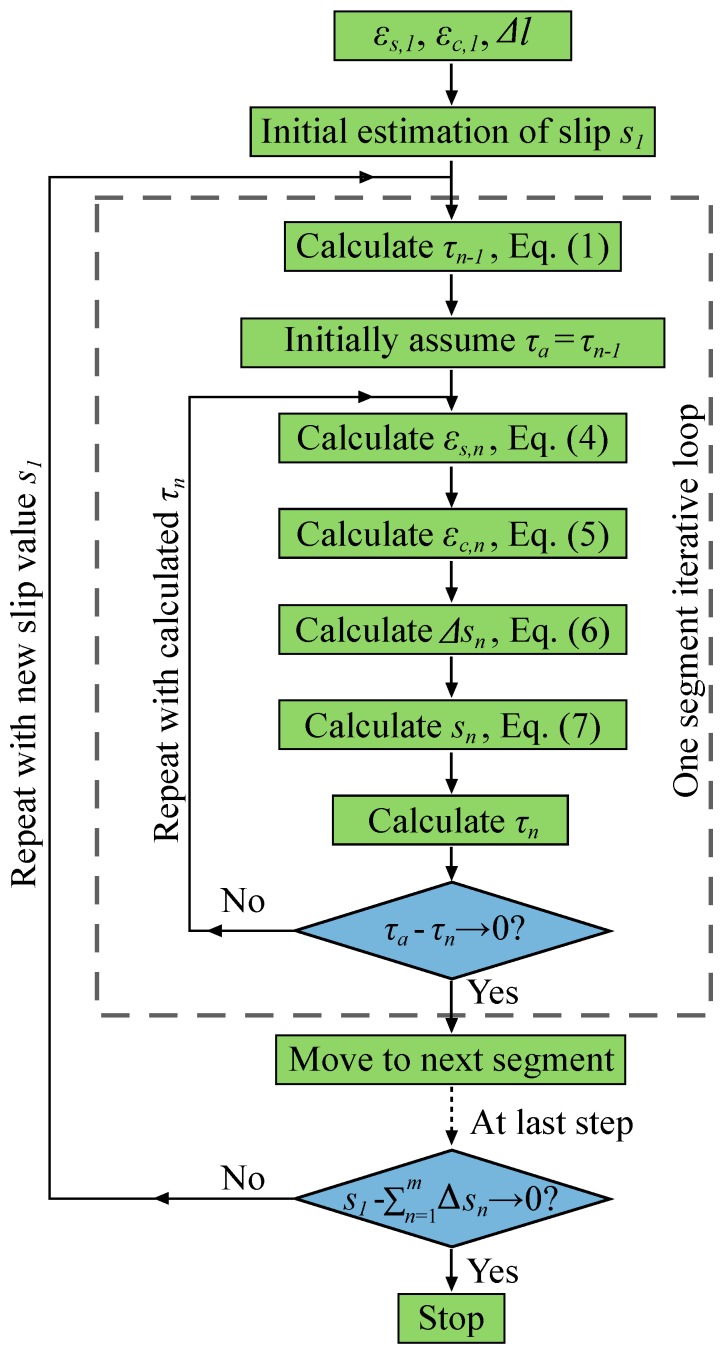
Flowchart of the iterative stress transfer procedure.

The general algorithm flowchart is presented in Figure 6.

**Figure 7:**
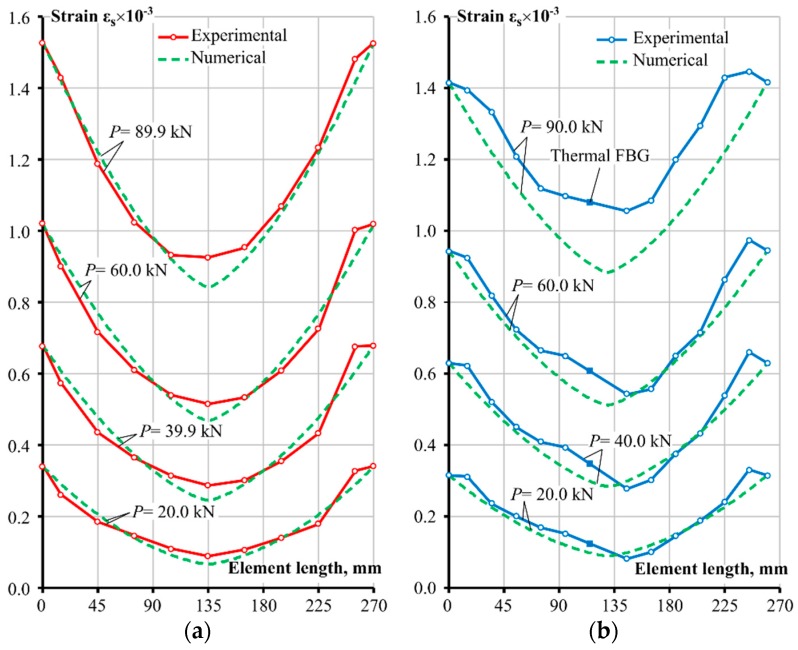
Predicted versus experimental reinforcement strain profiles: (**a**) Tensor strain gauge test and (**b**) FBG sensor test.

The stress transfer approach with the Model Code 2010 bond relationship as the basis had relatively accurate approximations of strain profiles in comparison with the experimental results, as shown in Figure 7. Good agreement was observed for all loading levels of the tensor strain gauge specimen, whereas the FBG began to diverge at the highest loading case, with remaining strain profiles being in relatively good agreement with the experimental ones as well. While the Model Code 2010 bond-slip relationship was able to provide relatively accurate strain distributions for the tested specimens reinforced with Ø20 mm bars, different diameter bars need to be investigated as well as more configurations of varying reinforcement ratios and bar sizes with multiple specimens.

**Figure 8:**
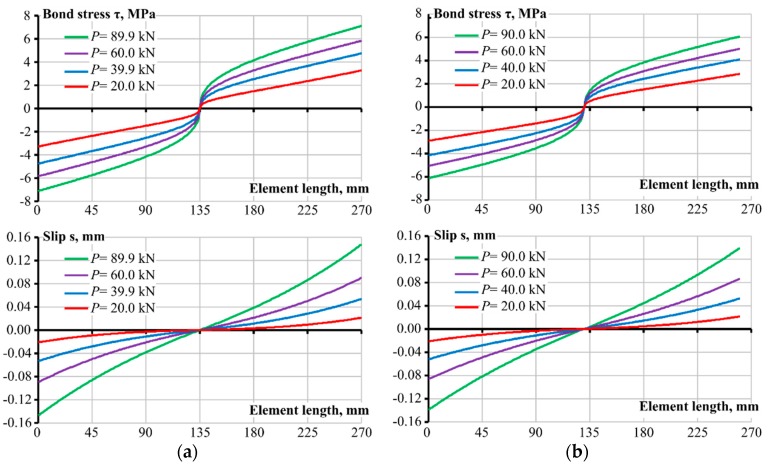
Estimated bond stress and slip distribution along the test specimens: (**a**) Strain gauge test and (**b**) FBG sensor test.

Figure 8 presents slip and bond stress distribution along the length of the experimental specimens. Maximal values of bond stress, *τ* = 7.1 MPa, and slip, *s* = 0.15 mm, were obtained. Both characteristics were well below the limit values, taken as the maximum bond stress, *τ*_max_ = 15.3 MPa, and the peak slip, *s*_0_ = 1 mm. The slip value, *s* = 0.15 mm, suggests that the mean crack width of an infinitely long RC member with the characteristics of the current test specimen would be *w* = 0.30 mm. The latter is based on the assumption that the length of the test specimen corresponds to the mean crack spacing. 

For bond investigation, ordinary pull-out tests are more common [25,26,27], however, these tests develop compressive stresses in the concrete as opposed to the real behaviour of the tensile zone of tensile or bending elements. Furthermore, the tests are carried out by pulling a reinforcement bar with an embedment length of 10 diameters, thus giving a highly averaged response. The ability of the double pull-out tests to evaluate the reinforcement strains within the bars makes an investigation of an element closer to its actual behaviour.

Regarding the experiments, the authors believe that both strain measuring approaches provide quite robust results when considering the limitations in the number of gauges installed. However, the comparatively high costs of the optical FBG strain gauge system and observed strain value fluctuations over the distance of the reinforcement bar require additional consideration when preparing the experimental program. Tensor strain gauges were employed for a significantly longer time than optical systems, hence their installation and data recording procedures were more robust and reliable, but installing the gauges inside the bar introduces more complexity over the FBG approach. As more attention must be given to how wiring is handled, precise milling of the bars and careful gluing of the two sides together, as well as the inability to provide very dense spacing of data points due to the large sizes of the tensor strain gauges. Whereas, the optical FBG system provides the ability to have denser gratings, hence more sensors to record data points from.

Although the above strain measurement techniques of the reinforcement bar embedded into concrete have limitations, in the authors’ view, it is the only currently available approach permitting adequate study of the bond-slip behaviour at the serviceability limit state. A bond-slip relationship developed from comprehensive test data would permit adequate analysis of such parameters, such as the crack spacing, crack width, and concrete and reinforcement strain distribution, between the cracks. 

Although the above tests are labour-intensive and time consuming, they are quite unique and are needed mainly for development of a comprehensive bond-slip law. This, in fact, means that a limited number of such tests is needed and the above shortcomings of the approach can be disregarded to achieve the main objective. Based on the results of the current study, the authors are starting a comprehensive experimental campaign to derive a new bond-slip law. 

It is important to note that alternative optical strain measuring techniques are gaining popularity and the attention of the researchers, namely, the optical frequency domain reflectometry approach for highly distributed sensing to record reinforcement values and provide more detailed strain variation profiles [28,29,30,31]. With technical possibilities, future research should investigate an equivalent specimen to this experimental study by the means of this or more advanced technology.

## 5. Conclusions

Two distinct experimental programs of tensile RC members were presented in this paper. The strain profiles of the reinforcement bars attained from the FBG optical strain gauge and ordinary tensor strain gauge experiments were used in a comparison. In addition, numerical calculations were carried out using the stress transfer approach to obtain the numerical strain variation along the reinforced concrete prism reinforcing bar.

The followings conclusions are drawn by the authors from this experimental and numerical study:

- The experimental techniques provided strain variations along the steel bar with reasonable accuracy and smoothness, however, the specimen strain results of the fibre Bragg grating optical gauge test displayed anomalies, particularly near the end of the specimen. The measured strains were larger than the strain value estimated for a bare bar assuming the experimental value of the modulus of elasticity of the bar. In addition, the FBG recordings were lacking consistency regarding the symmetry condition of the left and the right side of the member. However, the FBG approach is far less complex in preparing the specimen, as opposed to the tensor strain gauge experimental setup, which required careful milling, installation of gauges, and fastening of the bars back together. Moreover, the FBG technique can be used for smaller diameter bars, which would not be possible with the strain gauge approach due to a significant loss of section and lack of remaining sectional area to glue the two halves of the bar together. An option of a finer spacing of the sensors is another advantage of the FGB technique over the strain gauge approach.
- The observed behaviour in the strain profiles towards the ends of the reinforced concrete elements demands further investigation. Due to fluctuations in the strain values recorded by FBG sensors and the rather rare spacing between the sensors, it cannot be firmly asserted whether the phenomenon was related to the debonding effect proposed in previous research by the authors. The effect implies altered bond behaviour between the reinforcement and the concrete material surrounding it around the location of the cracks or elements’ ends.
- A comparison of the numerical results with appropriate experimental strain curves of the steel reinforcement showed that the Model Code 2010 bond-slip equation could provide sufficiently accurate results when coupled with the stress transfer approach for the Ø20 mm specimens. However, additional tests with different diameter bars and reinforcement ratios would help either affirm or negate the findings. Some discrepancy in numerical and experimental strain comparison appeared at the highest loading of the FBG specimen. The overall observed behaviour was parabolic, with steeper strain gradients at higher loading stages.
- The stress transfer approach can provide very comprehensive results of reinforcement and concrete strains, the bond stress and slip values over the entire distribution of the element, provided the implemented bond-slip model is accurate.

## Figures and Tables

**Table 1:** Specimen material and geometrical properties.

Test Setup	H × B × L, mm	Bar Diameter, mm	*A_s,ef_*, cm^2^	*f_y_*, MPa	*E_s_*, GPa	*f_cm_*, MPa	*E_cm_*, GPa
Strain gauge	150 × 150 × 270	Ø20	2.90	527	204	45.5	34.6
FBG sensor	150 × 150 × 260	Ø20	3.11	527	204	36.8	32.5

## References

[1] Balázs G.L., Bisch P., Borosnyói A., Burdet O., Burns C., Ceroni F., Cervenka V., Chiorino M.A., Debernardi P., Eckfeldt L. (2013). Design for SLS according to fib Model Code 2010. Struct. Concr..

[2] Farra B., Jaccoud J.P. Bond behaviour, tension stiffening and crack prediction of high strength concrete. Proceedings of the International Symposium Bond in Concrete.

[3] Beeby A.W. (2004). The influence of the parameter ϕ/ρeff on crack widths. Struct. Concr..

[4] Borosnyói A., Balázs G.L. (2005). Models for flexural cracking in concrete: The state of the art. Struct. Concr..

[5] Broms B.B. (1965). Crack width and crack spacing in reinforced concrete members. ACI J..

[6] Clark A.P. (1956). Cracking in reinforced concrete flexural members. J. Proc..

[7] Saliger R. (1936). High grade steel in reinforced concrete. Proceedings of the 2nd Congress of IABSE.

[8] Somayaji S., Shah S.P. (1981). Bond stress versus slip relationship and cracking response of tension members. J. Proc..

[9] Balazs G.L. (1993). Cracking analysis based on slip and bond stresses. Mat. J..

[10] Comité Européen de Normalisation (CEN) (2004). Eurocode 2: Design of Concrete Structures: Part 1-1: General Rules and Rules for Buildings.

[11] Fédération Internationale du Béton (FIB) (2010). Model Code 2010.

[12] Du Béton F.I. (2000). Bond of Reinforcement in Concrete: State-of-Art Report.

[13] Scott R.H., Gill P.A. (1987). Short-term distributions of strain and bond stress along tension reinforcement. Struct. Eng..

[14] Mains R.M. (1951). Measurement of the distribution of tensile and bond stresses along reinforcing bars. J. Proc. ACI J..

[15] Bresler B., Bertero V. (1968). Behavior of reinforced concrete under repeated load. J. Struct. Div..

[16] Houde J. (1974). Study of Force-Displacement Relationships for the Finite-Element Analysis of Reinforced Concrete. Ph.D. Thesis.

[17] Kankam C.K. (1997). Relationship of bond stress, steel stress, and slip in reinforced concrete. J. Struct. Eng..

[18] Wenkenbach I. (2011). Tension Stiffening in Reinforced Concrete Members with Large Diameter Reinforcement. Ph.D. Thesis.

[19] Masukawa J. (2012). Degradation of Shear Performance of Beams due to Bond Deterioration and Longitudinal bar Cutoffs. Ph.D. Thesis.

[20] Davis M.A., Bellemore D.G., Kersey A.D. (1997). Distributed fibre Bragg grating strain sensing in reinforced concrete structural components. Cem. Concr. Compos..

[21] Kenel A., Nellen P., Frank A., Marti P. (2005). Reinforcing steel strains measured by Bragg grating sensors. J. Mater. Civ. Eng..

[22] Kaklauskas G., Ramanauskas R., Jakubovskis R. (2017). Mean crack spacing modelling for RC tension elements. Eng. Struct..

[23] Li W., Xu C., Ho S.C.M., Wang B., Song G. (2017). Monitoring Concrete Deterioration Due to Reinforcement Corrosion by Integrating Acoustic Emission and FBG Strain Measurements. Sensors.

[24] Kaklauskas G. (2017). Crack model for RC members based on compatibility of stress-transfer and mean-strain approaches. J. Struct. Eng..

[25] Kim S.-W., Park W.-S., Jang Y.-I., Jang S.-J., Yun H.-D. (2017). Bonding Behavior of Deformed Steel Rebars in Sustainable Concrete Containing both Fine and Coarse Recycled Aggregates. Materials.

[26] Bouazaoui L., Li A. (2008). Analysis of steel/concrete interfacial shear stress by means of pull out test. Int. J. Adhes. Adhes..

[27] Dang C.N., Murray C.D., Floyd R.W., Hale W.M., Marti-Vargas J.R. (2014). Analysis of bond stress distribution for prestressing strand by Standard Test for Strand Bond. Eng. Struct..

[28] Bado M.F., Casas J.R., Barrias A. (2018). Performance of Rayleigh-Based Distributed Optical Fiber Sensors Bonded to Reinforcing Bars in Bending. Sensors.

[29] Ding Z., Wang C., Liu K., Jiang J., Yang D., Pan G., Pu Z., Liu T. (2018). Distributed Optical Fiber Sensors Based on Optical Frequency Domain Reflectometry: A review. Sensors.

[30] Barrias A., Casas J.R., Villalba S. (2017). Application study of embedded Rayleigh based Distributed Optical Fiber Sensors in concrete beams. Proc. Eng..

[31] Henault J.M., Quiertant M., Delepine-Lesoille S., Salin J., Moreau G., Taillade F., Benzarti K. (2012). Quantitative strain measurement and crack detection in RC structures using a truly distributed fiber optic sensing system. Constr. Build. Mater..

